# Carbohydrate mouth rinse: does it improve endurance exercise performance?

**DOI:** 10.1186/1475-2891-9-33

**Published:** 2010-08-27

**Authors:** Vitor de Salles Painelli, Humberto Nicastro, Antonio H Lancha

**Affiliations:** 1 Laboratory of Applied Nutrition and Metabolism, Department of Biodynamics of Human Movement, School of Physical Education and Sports, University of São Paulo, São Paulo, Brazil

## Abstract

It is well known that carbohydrate (CHO) supplementation can improve performance in endurance exercises through several mechanisms such as maintenance of glycemia and sparing endogenous glycogen as well as the possibility of a central nervous-system action. Some studies have emerged in recent years in order to test the hypothesis of ergogenic action via central nervous system. Recent studies have demonstrated that CHO mouth rinse can lead to improved performance of cyclists, and this may be associated with the activation of brain areas linked to motivation and reward. These findings have already been replicated in other endurance modalities, such as running. This alternative seems to be an attractive nutritional tool to improve endurance exercise performance.

## Background

Studies investigating the effects of carbohydrate (CHO) intake before and during exercise have accumulated since the beginning of the 20th century. The study of Krogh & Lindhardt [1] is considered one of the first to hypothesize and recognize the importance of CHO as an energy source for exercise. The authors demonstrated that subjects who underwent a high-CHO diet reported greater facility in accomplishing the proposed exercise (ergometer cycling and/or run) compared with those who consumed a high-fat diet, and this response was accompanied by higher rates of respiratory exchange during exercise. Later, in the mid 60's, using a muscle-biopsy technique, Bergstrom & Hultman [2,3] indicated for the first time the crucial role of muscle glycogen on endurance capacity (time to exhaustion), by demonstrating higher levels of muscle glycogen after consumption of high-CHO diet.

Since then, the ergogenic effects of CHO supplementation on endurance exercise performance have been consistently investigated. The mechanisms by which CHO supplementation promotes ergogenic effects may include both blood glucose and rates of CHO oxidation maintenance [4-6], a sparing effect on liver glycogen [7], stimulation of glycogen synthesis during low-intensity exercise [8] and/or exerting a possible stimulatory effect on the central nervous system (CNS) [9].

From the well-established mechanisms of CHO ergogenic action, it could be hypothesized that CHO supplementation would exert its ergogenic effect on long duration exercise, where the endogenous CHO could limit the performance at the latter stages of exercise [10,11]. However, CHO supplementation immediately before and during exercise of a shorter and more intense nature (>75% VO_2máx_, ≤1 hour) has also been shown to improve performance [12-14]. Possibly, this result could be related to a higher total CHO oxidation rate from the exogenous CHO, allowing high rates of energy expenditure in the latter stages of the exercise. Investigating this hypothesis, Carter et al. [15] demonstrated the contribution of infused CHO during a high-intensity exercise of 1-hour duration. Interestingly, higher RER values and plasma glucose concentrations in both placebo and CHO trials observed in this study suggest that endogenous CHO stores are not a limiting factor in high-intensity exercise, despite high muscle glucose uptake. Because of these results, the hypothesis of a CNS-mediated ergogenic action through oral and/or gastrointestinal glucose sensitive receptors became a strong target of investigation. Although few studies are available in the literature in the context of CHO mouth rinse and exercise performance, the data available so far point to an interesting dietary strategy to be used in certain sports (Table 1).

**Table 1:** Effects of carbohydrate mouth rinse on performance in endurance exercise

Reference	Type Of Activity/Exercise Protocol	Mouth Rinse Protocol	Nutritional Status	Performance Results
Carter et al [15]	Cycling/1 hour at 75% W_máx_	6,4% Malto (25 mL) every 12,5% of the trial completed	4-hour fasting	- 2,9% TT/+ 2,7% W
Whitham & McKinney [24]	Running/"self-paced" 45 min	6% Malto (200 mL) before and every 6 min of trial	4-hour fasting after standard diet	No difference
Rollo et al [23]	Running/10 min at 60% VO_2máx _- 30 min according to "hard pace" RPE in Borg Scale	6% Glucose (25 mL) before and every 5 min of trial	Overnight Fasting	+ 1,7% distance
Pottier et al [23]	Cycling/1 hour at 75% W_máx_	6% CHO-electrolyte solution (1.5 mL/kg b.w.) before and every 12,5% of the trial completed	3-hour fasting	- 3,7% TT/+ 3,3% W
Chambers et al [20]	Cycling/1 hour at 75% W_máx_	6,4% Malto (25 mL) before and every 12,5% of the trial completed	6-hour fasting/overnight fasting	- 2,9% TT/+ 2,7% W
Beelen et al [25]	Cycling/1 hour at 75% W_máx_	6,4% Malto (25 mL) before and every 12,5% of the trial completed	2-hour fasting after standard diet	No difference

Malto - Maltodextrin; RPE - Rate of Perceived Exertion; TT - Time Trial; VO_2máx _- Maximal Aerobic Capacity; W - Average Power Output; W_máx _- Maximal Power Output

### Carbohydrate mouth rinse and endurance exercise performance

Jeukendrup's group was the first to innovate in this direction. They observed a 2.9% decrease in a time-trial test at 75% Wmáx lasting about 1 hour, rinsing the subjects' mouths for 5 seconds with a CHO solution (25 ml containing 6.4% maltodextrin), every 12.5% of the trial completed [9]. The above finding strengthened the hypothesis of the possible central effect of CHO on exercise performance and that this could be acting through activation of receptors linked to the brain.

Seeking mechanisms to elucidate the above results, Chambers et al. [16] evaluated the effect of CHO rinsing (a solution containing glucose and/or maltodextrin) in a cycle time-trial of 1 hour. The authors used magnetic resonance imaging (MRI) method in a second set of experiments to identify possible areas of brain activated with CHO mouth rinse. Trained cyclists performed a time trial (75% of maximal work for 1 hour) in 3 different situations: glucose, maltodextrin, or placebo mouth rinse with a washout period of at least 3 days. All situations were performed after 6-hour fasting. An artificial sweetener was added to the solutions in order to reduce sensory clues. The mouth rinse protocol lasted ~10 seconds and the solution was rinsed every 12.5% of the trial completed. The results showed that a solution with 6.4% glucose and/or maltodextrin produced an improvement of 2-3% in the time trial and the average power when compared to placebo. There were no differences between the solutions containing different types of CHO. MRI evaluations showed that CHO mouth rinse activated supraspinal pathways of the brain related to motivation and reward during the exercise. Brain activation was similar to the solutions of glucose and maltodextrin, while the placebo solution did not produce the activation of these areas. These results demonstrate the important role of CHO mouth rinse on exercise performance, which could be an interesting strategy being used by athletes who suffer from gastrointestinal discomfort, for example, due to the CHO ingestion during the exercise.

Gastrointestinal discomfort is an adverse effect of CHO intake more often observed during running compared to cycling [17] due to stress of abdominal organs caused by the jumping movements of the exercise [18]. Since CHO mouth rinse could be implemented in a running routine as an alternative tool to avoid such a collateral effect, Rollo et al. [19] evaluated the influence of a 6% CHO mouth rinse administered every 5-minute intervals on the performance of recreational runners during a 30-minute treadmill race at a speed equal to a rate of perceived exertion of 15 on Borg's Scale [20]. The results showed that the total distance covered after the CHO mouth rinse was higher than the placebo. This is due to the fact that during the first 5 minutes of the test the mouth rinse provided lower levels of perceived exertion and, consequently, a higher speed was achieved. Although the study was not composed of a group that drank the solution instead of mouth rinsing, one may speculate that this strategy could have prevented the occurrence of symptoms related to gastrointestinal discomfort.

Thinking about the lack of results in literature comparing the afore mentioned point, Pottier et al [21] made a direct comparison between rinsing and drinking a sweetened 6% CHO-electrolyte solution (CES) during a high-intensity exercise (75% W_máx_) of moderate duration (~1 hour). To accomplish this objective, they submitted 12 endurance-trained triathletes under four experimental conditions: rinsing CES, rinsing placebo, ingesting CES and ingesting placebo. The solutions were administered every 12.5% of the trial completed. Interestingly, the authors showed that rinsing the mouth with a CES led to a 3.7% improvement in performance compared to an electrolyte-containing placebo. This could be explained by the higher mean power output throughout the trial observed in the CES rinsing treatment and by the non-altered rate of perceived exertion between treatments. Therefore, the diminished subjective perception of a given exercise intensity allowed subjects to produce more power output for the same degree of discomfort. But probably, the most inquisitive finding of this study is related to the fact that mouth rinsing, but not the ingestion of a CES, resulted in an improved performance. Pottier et al [21] suggested that perhaps this surprising finding may be related to the duration the beverages were kept in the oral cavity, thus probably increasing brain stimulation. However, the physiological advantage gained with the mouth rinse over the intake could be attributed to a reduced requirement of blood supply and energy cost by the gastrointestinal tract to digest and absorb the CHO (which are eventually unnecessary to sustain the exercise of relatively short duration).

One detail that certainly deserves to be discussed in a brief commentary refers to the fact that both Rollo et al. [19] and Pottier et al. [21] used a sweet drink with simple sugars and sweetened placebo (to mask the sweet CHO taste) although the majority of the studies is done with non-sweet maltodextrine solutions, and even so, these studies showed an improvement in performance after rinsing the mouth with CHO. This result raises an intriguing question: how can the human mouth distinguish between a sweet non-caloric and a sweet caloric drink? The mammalian sweet taste receptor combines two G-protein-coupled receptors, T1R2 and T1R3, which respond to both natural sugars and artificial sweeteners [22]. These taste receptor cells found primarily on the tongue are innervated by afferent fibres that transmit information to taste regions in the cortex via the thalamus [23]. Recent work using transgenic mice that lack the T1R3 protein suggests that natural caloric sugars activate taste afferents differently from non-caloric artificial sweeteners [24,25]. T1R3-knock-out (KO) mice showed no behavioural attraction to artificial sweeteners. Yet there was only a modest reduction in preference to caloric sugars [25] and T1R3-KO mice still had a detectable gustatory nerve response to natural sugar. More recently, Delay et al. [26] reported that the detection threshold for sucrose was indistinguishable between T1R3-KO and wild-type mice. Although the effects of T1R3 deletion have not been tested on an exercise performance, these results indicate that there are T1R3-independent taste receptors for natural carbohydrates in mice. It should be noted that there are no human studies about this subject.

Although several studies have reported an improved exercise performance with mouth rinse, Whitham & McKinney [27] did not observe the same ergogenic effect of rinsing a 6% maltodextrin solution in a running protocol with the first 15 minutes at 65% VO2max followed by 45 minutes at a speed self-selected by runners. One possible reason to explain the difference among the results from Rollo et al [19] and Whitham & McKinney [27] studies mentioned above refers to the runners' nutritional status of each study. While the subjects from Rollo's study [19] arrived at the laboratory after an overnight fasting, Whitham & McKinney's study [27] standardized the subjects's diet and asked them to consume it 4 hours before the experimental protocol. It is also worth mentioning that the cyclists from Chambers et al [16] conducted performance tests after 6-hour fasting. Still, an improved performance was observed in the athletes from Pottier et al [21] and Carter et al [9] after just 3 and 4- hour fasting, respectively. Thus, it is hard to speculate that the potential stimulatory effect of glucose in the mouth may be of considerable impact only under conditions where the liver's glycogen may be depleted.

In this context, Beelen et al [28] reproduced the same experimental design as Carter et al [9], but allowed cyclists to ingest a standard meal 2 hours before 75% Wmáx time trial for about 1 hour, and found no ergogenic effects with 6.4% maltodextrin solution mouth rinsing. One possible explanation for these results lies in recent studies using animal models where the loose-patch technique for recording from taste buds in situ has provided important information about transduction mechanisms in mammals for sweeteners [29]. The technique consists of recording action currents, reflecting taste cell action potentials, from single fungiform taste buds in situ. Previous studies have shown that responses to sweeteners in any single taste bud are reliable and repeatable for up to periods of 2 h [29,30]. From this point, it is possible to infer that pre-exercise feeding may influence the brain responses to an oral CHO stimulus during a subsequent exercise because it is likely that the activation of brain regions associated with feeding and reward, such as the orbitofrontal cortex and striatum, are modulated by homeostatic regulation as well as the current physiological state of the body [31]. In this context, it is possible to suppose that the nutritional status has a direct bearing on the ergogenic effect provided by a CHO mouth rinse. Therefore, it would be interesting to see if the results are reproducible in practical situations, for example, in the postprandial state, when the athletes are used to eating CHO-rich meals before competition.

## Summary

Given the results described, the literature points to a possible new interesting nutritional strategy to be used in order to improve high-intensity endurance exercise performance. In addition, CHO mouth rinse may have an important role on the mechanisms of central fatigue that so far has not been evaluated. It should be emphasized that even studies where liver glycogen stocks were available for the maintenance of energy during exercise, ergogenic benefits have been found. Therefore, the external validity of this nutritional strategy is not questionable because the positive results were found both in models with or whithout acute energy restriction. However, more controlled studies about the real function of oral CHO receptors in different nutritional states are still needed. Furthermore, future studies should test if this same nutritional strategy could improve exercise performance in other sports, such as those related to strength and power.

## Competing interests

The authors declare that they have no competing interests.

## Authors' contributions

VSP: participated in the data acquisition, analysis and interpretation, drafted the manuscript and reviewed the manuscript before submission; HN: participated in the design of the manuscript, data analysis, helped to draft the manuscript and significantly reviewed the manuscript before submission; AHL Jr: conceived the idea of the manuscript, and participated in its design and coordination and helped to draft the manuscript; All authors read and approved the final manuscript.
